# A novel graph theoretical approach for modeling microbiomes and inferring microbial ecological relationships

**DOI:** 10.1186/s12864-019-6288-7

**Published:** 2019-12-20

**Authors:** Suyeon Kim, Ishwor Thapa, Ling Zhang, Hesham Ali

**Affiliations:** 1 School of Interdisciplinary Informatics, University of Nebraska at Omaha, Omaha, 68182 NE USA; 2 College of Information Science and Technology, University of Nebraska at Omaha, Omaha, 68182 NE USA

**Keywords:** Microbiomes, Graph theoretic models, Data integration, Split graphs

## Abstract

**Background:**

Microbiomes play vital roles in shaping environments and stabilize them based on their compositions and inter-species relationships among its species. Variations in microbial properties have been reported to have significant impact on their host environment. For example, variants in gut microbiomes have been reported to be associated with several chronic conditions, such as inflammatory disease and irritable bowel syndrome. However, how microbial bacteria contribute to pathogenesis still remains unclear and major research questions in this domain remain unanswered.

**Methods:**

We propose a split graph model to represent the composition and interactions of a given microbiome. We used metagenomes from Korean populations in this study. The dataset consists of three different types of samples, viz. mucosal tissue and stool from Crohn’s disease patients and stool from healthy individuals. We use the split graph model to analyze the impact of microbial compositions on various host phenotypes. Utilizing the graph model, we have developed a pipeline that integrates genomic information and pathway analysis to characterize both critical informative components of inter-bacterial correlations and associations between bacterial taxa and various metabolic pathways.

**Results:**

The obtained results highlight the importance of the microbial communities and their inter-relationships and show how these microbial structures are correlated with Crohn’s disease. We show that there are significant positive associations between detected taxonomic biomarkers as well as multiple functional modules in the split graph of mucosal tissue samples from CD patients. Bacteria *Moraxellaceae* and *Pseudomonadaceae* were detected as taxonomic biomarkers in CD groups. Higher abundance of these bacteria have been reported in previous study and several metabolic pathways associated with these bacteria were characterized in CD samples.

**Conclusions:**

The proposed pipeline provides a new way to approach the analysis of complex microbiomes. The results obtained from this study show great potential in unraveling mechansims in complex biological systems to understand how various components in such complex environments are associated with critical biological functions.

## Background

The widespread use of high throughput sequencing technologies and its declining cost provide great opportunity to explore advanced properties of complex microbiomes and study the impact of their properties on the health of organisms associated with their environments. A variety of techniques have been applied to describe the composition of microbial communities, mainly through 16s rRNA sequencing. For example, using 16s rRNA data, recent findings show that variations and interactions between intestinal microbiota and their host environments play a significant role in human health and disease [1–3]. Such interactions take different shapes and forms such as mutualism, competition, and parasitism. These alterations correspond to changes in the development and maintenance of mucosal homeostasis and the loss of that function contributes to intestinal inflammation [4–6]. For example, microbiome studies have linked inflammatory bowel disease (IBD) to alterations in both the microbial communities of the human gut and the intestinal immune system [7, 8]. However, such studies remain in early stages and there is a need to fully understand how microbial interactions occur at the community level, and how these interactions may play a role in human health and susceptibility to suffer from various diseases.

With the availability of new microbiome data, recent research efforts have been aimed at inferring microbial ecological interactions from microbial abundances as well as observing correlations between microbes and disease status. The majority of such efforts rely on various statistical approaches, including classical correlation analysis, Sparse Correlations for compositional data (SparCC), and SpiecEasi (SParse InversE Covaraince Estimation for Ecological ASsociation Inference), to study the network of microbial interactions [9, 10]. In addition, due to the availability of large sets of data, different machine learning methods have been utilized to understand how microbes interact with each other to form functional communities and potentially affect the health of organisms in their environments. Basic ideas for utilizing co-occurrence analysis, based on network inference to capture significant co-occurrence relationships among the microbial abundances, have been used in multiple microbial studies [11]. For example, *Mandakovic et al.* investigated how co-occurring microbial communities correspond to environmental factors using CoNet application [12]. This method was able to infer microbial networks based on different statistical measures using microbial abundances. Such networks can also represent relationships between microbes and ecological factors. All such studies, however, remain in their early stages. This is primarily due to the complexity and the dynamic nature of microbial ecosystems [13, 14]. There is also a lack of a robust model that allows researchers to model different types of relationships associated with complex microbiomes. In addition, there is a need for an integrated bioinformatics pipeline that quantifies microbiome parameters at multiple taxonomy levels and characterizes metabolic functional features and their associations to microbes. Such pipelines would be critical in understanding significant variations in the microbial compositions of healthy individuals compared to those with certain conditions such as IBD. Recognizing this complexity, a systems biology approach would attempt to model not only the interactions between microbial communities within a microbiome, but also how those interactions impact the health and functionality of organisms living in associated environments in an expanded and holistic context.

In this study, we explore the use of graph-theoretic approaches to properly address the complexities associated with studying complex microbiome environments. We present a split graph model to identify bacteria-bacteria and bacteria-bacterial metabolic functional relationships in different host health statuses. It takes advantage of the properties of this special class of graphs, including the fact that edges in such graphs are divided into two distinct groups of edges, in order to represent relationships within components of a given microbiome, as well as represent relationships between one or more microbial components and phenotypes of organisms in its environment. An earlier version of the model was used to identify the correlation between the bacterial abundance in different types of fish and gut locations with a variety of fish phenotypes [15].

This approach attempts to extract critical types of relationships associated with microbiome. The graph model is designed to specifically identify elements in microbiome that have significant impact on key biological functions or pathways. It allows us to better understand their impact individually and as functional groups as well as identify the inter-relationships of microbiome and their association with the functional pathways. Moreover, we can explore each microbial/functional biomarker further. We intend to integrate different types of data such as microbiome abundance levels, co-occurrence and metabolic functional information in order to accurately model the complex microbiome environments. An important goal of this study is to develop an advanced bioinformatics pipeline for metagenomics studies that highlights the bacteria-bacteria and bacteria-bacterial metabolic pathways in the microbial community of Crohn’s disease (CD) using the graph model. We validate our findings both using linear discriminant analysis (LDA) effect size (LEfSe) to determine the taxonomic levels or functions to differentiate between healthy and CD groups and by referring to published literature in this domain.

## Methods

In this section, we first describe the split graph model in detail and explain all the steps carried out in this study. The overall pipeline consists of two dependent parts to (a) create independent networks of inter-correlations (bacteria-bacteria) and external-associations (bacteria-metabolic functional pathways) using microbiome abundance data in conjunction with genomic information from these microbes and (b) to obtain split graphs from these networks.

### The split graph model

A ‘split graph’ is a graph G = (V, E), in which the set of vertices can be partitioned into two disjoint sets; an independent set (I) and a clique (Q), where V =I ∪ Q [16, 17]. In a given graph G, a clique or a complete subgraph is defined as a set of nodes Q in which every node is adjacent to every other node in Q. An independent set or an empty subgraph is a set of nodes I, where there are no relationships (or edges) between any pair of nodes in I. E represents two sets of edges. Edges that connect nodes in the clique Q can be referred to as clique edges and the edges connecting nodes in Q to nodes in I are defined as cross edges.

**Fig. 1:**
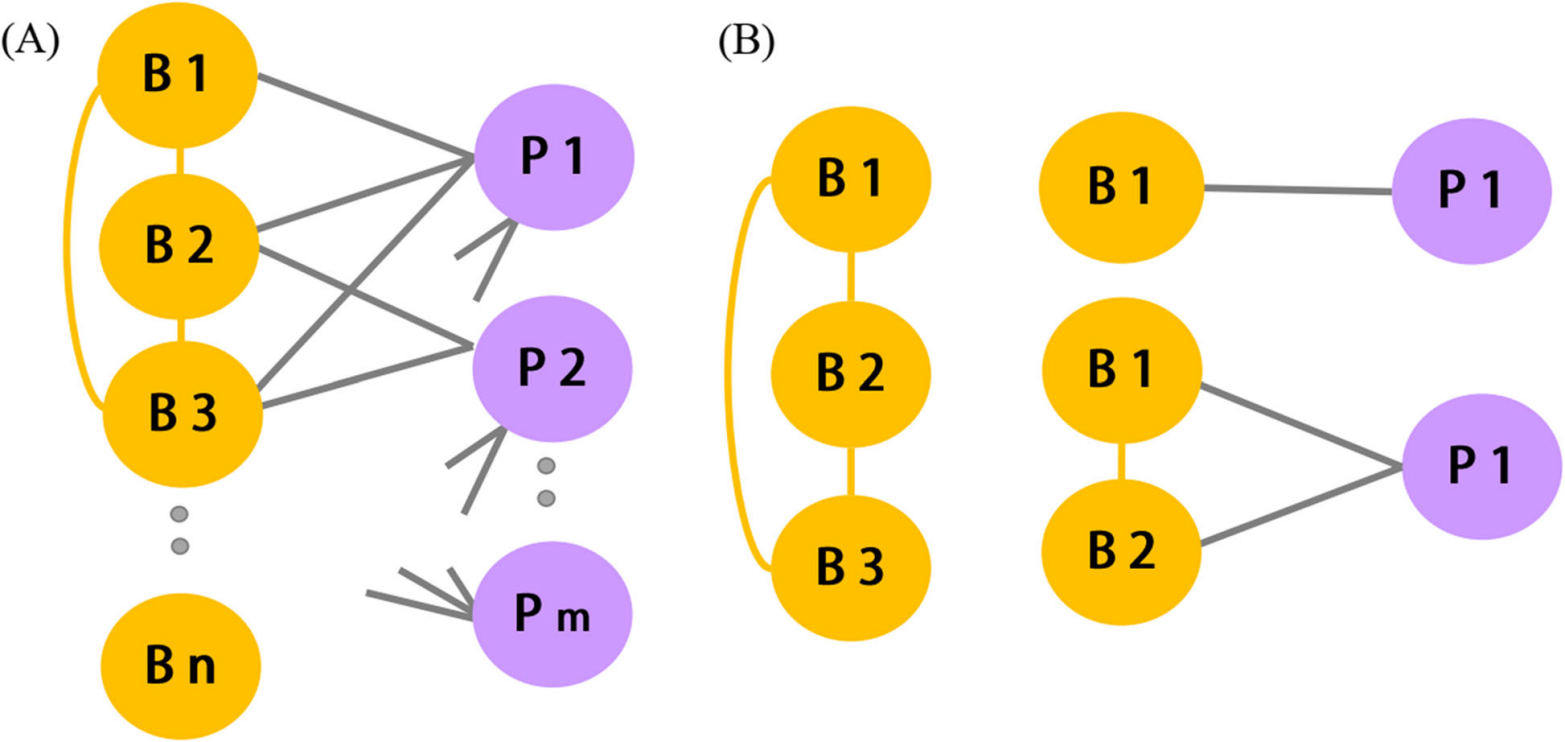
**a** The split graph model capturing two relationships, (i) inter-bacterial and (ii) bacteria and metabolic functions. Two different colors on the edges represent different relationships. **b** Multiple examples of clique model

We propose the use of split graphs since they can efficiently model the microbiome composition and its impact on its associated organisms. We represent the components of the microbiome as the nodes of the clique in the split graph. Similarly, the phenotypes or functional pathways that some bacteria belong to are modeled by the nodes in the independent set in the graph. The clique edges represent the interactions/relationships among the microbial components. A cross edge corresponds to the relationship between a microbial element and a phenotype. An example of split graph is shown in Fig. 1a. The nodes with yellow circles represent microbes (bacteria). The edges between these bacteria signify that they are highly correlated to each other (inter-relationship) and form a clique. The nodes with purple circles represent the phenotypes of organisms in associated environments, and the cross edges between one or more bacterial components and its phenotypes represent the external relationship.

We can use the weight on each edge to model different types of relationships such as co-occurrences or possible interactions/correlations. To detect robust associations between entities, we explored both co-occurrence patterns and correlations. Note that a clique in such graphs may contain at most one node from the independent set. Either a high-weighted clique in the graph may correspond to a set of high correlated/co-occurring microbial components or it may correspond to a set of highly correlated components along with a phenotype/pathway from the independent set. We show examples of such cliques in Fig. 1b. For example, the three components of a microbiome form a clique in the left hand side of Fig. 1b indicating that they highly co-exist in their environments. On the other hand, in the right hand side of Fig. 1b, one or two components of the microbiome in the clique have high correlations with a phenotype. Both types of cliques are considered while obtaining the split graph. This model makes it possible to extract different types of information based on the nature and structure of the input data.

Note that the independence of the nodes representing phenotypes in the model is intentional. Even if there are some dependencies among them, that would not have an effect on the information we are trying to extract from the model or on addressing the key research question, which is: how to identify elements or subgroups of elements in a microbiome that have a significant impact of a specific phenotype or a key function/pathway. Elements or groups of elements may impact more than one phenotype, but we will still be able to obtain such information from this model without looking at the inter-relationships among them. With the independence of the nodes, a highly-weighted maximal clique in the graph corresponds exactly to one module that contained one element or highly correlated elements from the microbiome composition and exactly one phenotype. Each such module is directly related to the question we are asking or the information we are trying to extract. Hence, the extracted information from the model is represented in terms of the well-known maximum weighted clique property.

### Data processing of 16S rRNA gene sequence datasets

We obtained 55 publicly available 16S rRNA datasets from NCBI SRA database with project accession number SRP039586. This data sets consist of three different biological samples: 36 mucosal tissue samples from Crohn’s disease patients (CDT), 10 stool samples from Crohn’s disease patients (CDS), and nine stool samples from healthy individuals (HCS). Quantitative Insight into Microbial Ecology (QIIME) bioinformatics pipeline is used for 16S rRNA sequence-based microbial community analysis [18]. While using this pipeline, the similarity threshold value of 97% was selected to cluster operational taxonomic units (OTUs) and the microbial classification was performed with reference to the Greengenes database [18, 19].

### Metagenome prediction and metabolic reconstruction of 16S rRNA datasets

The PICURST v1.1.0 software was used to predict metagenomes [14]. For the first step, the OTU table obtained in the previous step is normalized by dividing each OTU by its known 16S rRNA gene copy number abundance using the *normalize_by_copy_number.py* script. Employing the *predict_metagenomes.py* script, this normalized OTU table was used to predict KEGG Ortholog (KO) functional profiles of microbial communities [14]. For the final step, we obtained a table of annotated KO abundances for each metagenome sample in the OTU table using *metagenome_contributions.py* script. The built-in algorithm allows to link OTUs from a phylogenetic tree of 16S rRNA gene sequences to its gene contents. HuMAnN2 pipeline was utilized to reconstruct KEGG pathways from predicted KO functional profiles.

### Detection of taxonomic and metagenomics biomarkers

Linear discriminant analysis effect size (LEfSe) tool was used to identify the most biologically informative features, such as taxa composition and functional metabolic pathways, in three different groups (CDT, CDS, and HCS). It comprises of non-parametric Kruskal-Wallis (KW) test to explore differentially abundant features and LDA analysis to estimate the effect size between the comparison groups. Default statistical parameters of alpha = 0.05 and LDA score 2.0 were used for this analysis.

### Network construction and Split graph analysis

*1) Detection of inter-bacterial associations* We assessed the bacterial associations that reveal patterns in co-occurrence of microbes within each biological samples (CDT, CDS, and HCS). The associations for every pair of microbial species were statistically calculated using a non-parametric test of Spearman’s rank correlation analysis. Robust co-occurrence patterns, with the Spearman’s correlation coefficient (rho) >0.6 and the false-discovery rate (fdr) adjusted *p*-value <0.05, were identified. All of the analyses were carried out in the R environment.

**Fig. 2:**
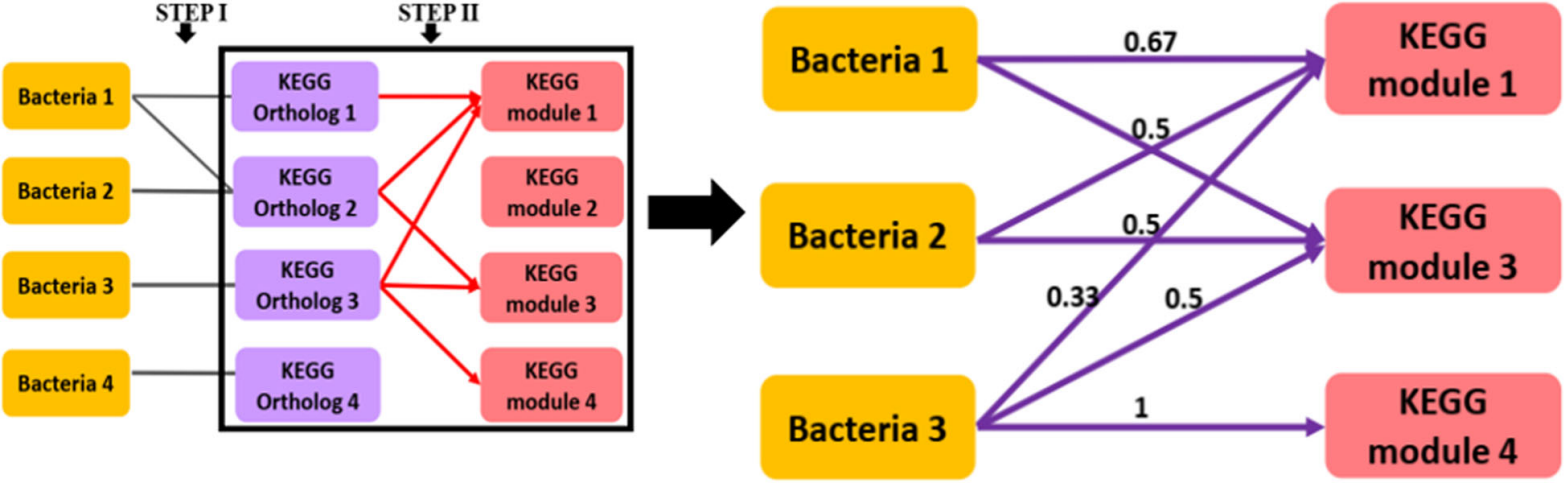
Overall framework to identify associations between bacterial taxa and their microbial pathways (Left). Calculation of proportion of KOs between bacterial taxa and KEGG module (Right)

*2) Detection of associations between bacterial taxa and bacterial metabolic pathways* This is a two-step process to identify associations between bacterial taxa and bacterial metabolic pathways. In the initial step, the association between the abundance of bacterial taxa observed in co-occurrence patterns and KEGG orthologues (KO) is estimated. Statistically significant associations were inferred with correlation coefficient (rho) >0.6 and adjusted *p*-value >0.05. The *p*-values were adjusted using the FDR correction in the R environment. In the subsequent step, the association between a bacterial taxon and a metabolic pathway was estimated as the ratio of KOs that are correlated to the bacteria to that of the total number of KOs in the KEGG modules. KEGG module information is obtained from the KEGG database using KEGG REST API for all the KOs [20]. Each KEGG module consists of many KOs as represented by the red edges in Fig. 2. Hence, for the $\phantom {\dot {i}\!}j^{\text {th}}$ Bacteria (*B*_*j*_) and the $\phantom {\dot {i}\!}i^{\text {th}}$ Module (*M*_*i*_):

1 $$ {Density}_{j}^{i} = \frac{Number\, of\, KO\, in\, M_{i}\, correlated\, with\, B_{j}}{Total\, number\, of\, KO\, in\, M_{i}}  $$

*3) Construct the network and build the corresponding split graph* We applied network-based analysis and split graph model to identify high-weighted maximal cliques that are both critical informative components of inter-bacteria correlations and association between bacterial taxa and bacterial metabolic pathways. Two distinct relationships are integrated in each three environments, including CDT, CDS, and HCS. The split graph containing two disjoint sets of nodes viz. correlated microbial communities and the microbial metabolic pathways were obtained. The critical components, high-weighted maximal cliques, were extracted from the split graph. These split graphs were visualized in the open-source Cytoscape v.3.4.0 software [21]. To elucidate association between clique and microbial metabolic pathways (density >0.6), the proportion of KOs over all possible KOs for each KEGG module (referred to as density here after) was used as weights for the cross edges (density >0.6).

### Comparing the difference of two population proportions

The final step of this pipeline involves comparison of proportion of correlated edges from diseased groups (CDS and CDT) that have a common ancestor. A two-proportion Z-statistics was used to analyze the test of significance difference of two population proportions (See Eq. 1). This statistics test the null hypothesis that the proportion of number of correlated edges with a common ancestor is equal across the groups.

2 $$ z = \frac{\hat{p1} - \hat{p2}}{\sqrt{\hat{p}(1-\hat{p})\left(\frac{1}{n1} + \frac{1}{n2}\right)}}  $$

## Results

### Detection of taxonomic biomarkers

**Fig. 3:**
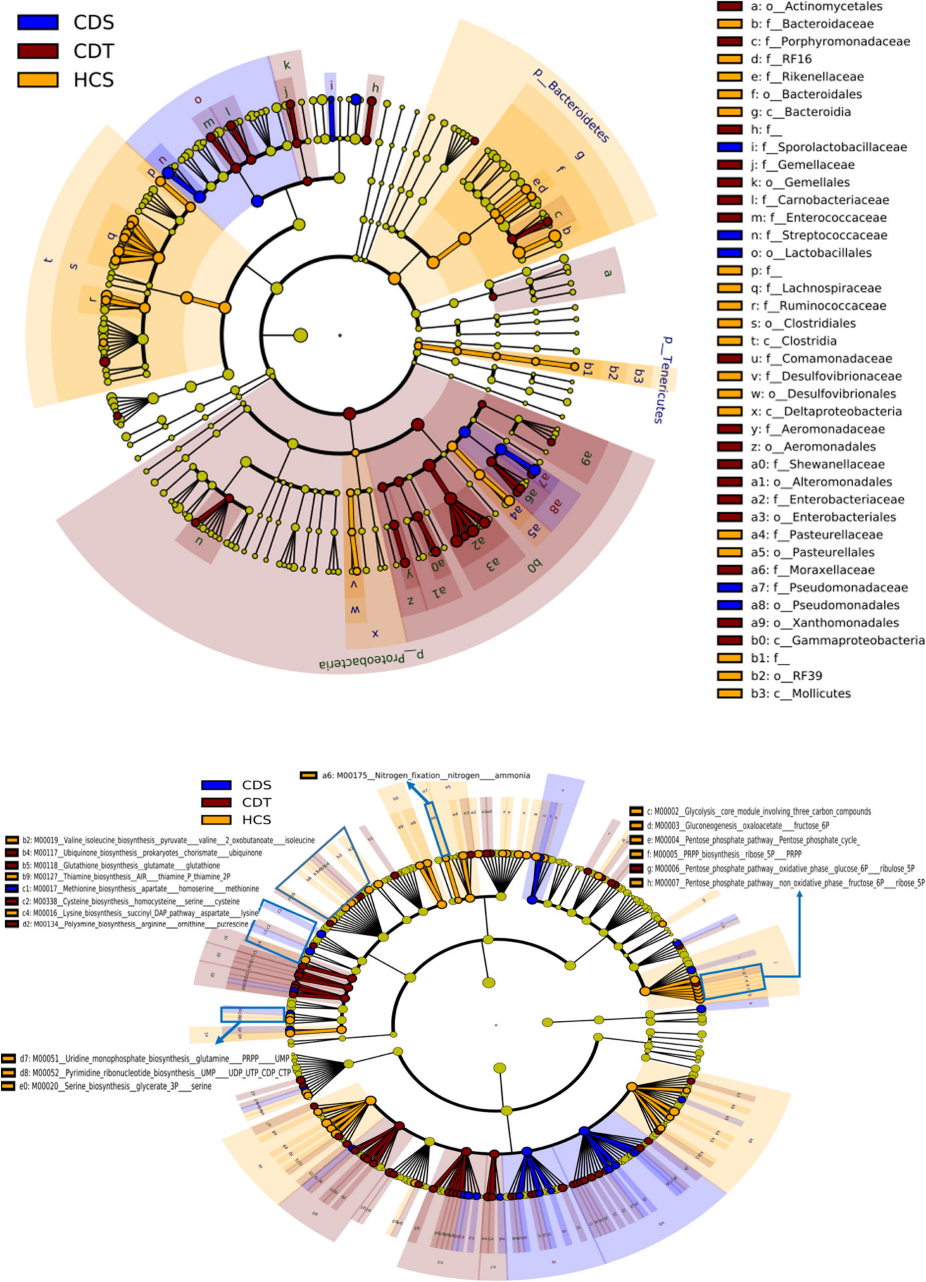
Cladograms generated from LEfSe for biomarker detection in taxonomic (top) and metabolic function pathways (bottom)

To identify core candidate microbiota biomarkers that are present in Crohn’s disease and healthy samples, a cladogram was constructed to demonstrate relative abundance of bacteria. Using LEfSe tool, we identified 40 differential abundant microbial taxonomic features in control samples, stool samples and mucosal tissue samples from CD. Small circle on the cladogram ring represents a taxonomic rank, which has different abundance values among the groups based on the LDA scores. All detected microbial taxonomic features can be presented in cladogram highlighting significant differences across three types of samples (See Fig. 3 (top)). We specifically discuss the results from family and genera biomarkers. The LEfSe analysis found *Streptococcaceae, Lactobacillales*, and *Pseudomonadaceae* are differentially abundant in the CDS, whereas *Porphyromonadaceae, Shewanellaceae*, and *Enterobacteriaceae* are differentially abundant in CDT. *Bacteroidaceae, Lachnospiraceae, Rikenellaceae*, and *Ruminococcaceae* were identified as taxonomic biomarkers for healthy individuals.

### Detection of metabolic functional biomarkers

In addition to microbial composition, we also compared differentially abundant functional and metabolic characteristics in three microbial samples. Figure 3 (bottom) highlights 135 differentially abundant functional modules detected in the microbial communities corresponding to CDT, CDS and HCS. While various microbial metabolic functions are carried out throughout the human microbiome, specific subsets of this functionality could be enriched in different types of samples. The LEfSe tool highlights these specific metabolic features (KEGG modules) as shown in Fig. 3 (bottom). Modules such as biosynthesis of lysine (M00016), and UMP (M00051) were differentially enriched in healthy control samples. We also found that the glutathione biosynthesis (M00118), metabolism of the sulfur-containing amino acids cysteine (M00338), and methionine biosynthesis (M00017) were significantly enriched in CDT. In addition, several other modules essential for basic life activities of prokaryotic cells, such as central carbohydrate metabolism (M00002-M00007) and amino acid metabolism (M00018, M00019, M00020, M00118 and M00338) are highlighted in the cladogram. These results exclusively show that the specific metabolic modules are enriched in distinct biological samples.

### Detection of bacterial interactions

We explored the inter-bacterial association networks at the family and genus level in three environments (CDT, CDS, and HCS). Table 1 and Additional file 1 present the results of the positive and negative associations among bacteria by a Spearman’s correlation approach. Less number of associations were identified in stool samples from Crohn’s disease patients and healthy individuals as opposed to mucosal tissues from Crohn’s disease samples.

**Table 1:** Inter-bacteria correlations in all sample groups

	Taxonomic clade	Taxonomic clade	R^2^
CDS	*f__Bacteroidaceae*	*f__Lachnospiraceae*	0.94
	*f__Aerococcaceae*	*f__Fusobacteriaceae*	0.98
HCS	*f__Prevotellaceae*	*f__RF16*	0.98
	*f__Bacillaceae*	*f__Staphylococcaceae*	0.98
	*f__Rikenellaceae*	*f__Ruminococcaceae*	0.97
CDT	*f__Aeromonadaceae*	*f__Shewanellaceae*	0.81
	*f__BA059*	*f__Syntrophobacteraceae*	0.72
	*f__Planococcaceae*	*f__Gallionellaceae*	0.72
	*f__Porphyromonadaceae*	*f__Pseudomonadaceae*	0.71
	*f__Carnobacteriaceae*	*f__Streptococcaceae*	0.70
	*f__Moraxellaceae*	*f__Pseudomonadaceae*	0.68
	*f__Microbacteriaceae*	*f__Spirochaetaceae*	0.68
	*f__BA059*	*f__Gallionellaceae*	0.68
	*f__Peptococcaceae*	*f__Alteromonadaceae*	0.68
	*f__Peptococcaceae*	*f__Sinobacteraceae*	0.68
	*f__Nitrospiraceae*	*f__Syntrophobacteraceae*	0.68
	*f__Procabacteriaceae*	*f__Halomonadaceae*	0.68
	*f__Veillonellaceae*	*f__Pseudomonadaceae*	-0.66
	*f__Porphyromonadaceae*	*f__Shewanellaceae*	0.66

In mucosal tissues from Crohn’s disease patients, 13 positive with one negative relationships were recognized between bacterial families. A strong positive correlation between *Aeromonadaceae* and *Shewanellaceae* was observed in Table 1 and both have common ancestor in their evolutionary lineages. There were also strong positive and negative associations between bacterial genera in CDT and CDS. Like CDT, significantly strong positive interactions were observed in CDS and healthy individual samples. All observed inter-bacterial associations at the genus level have shown high correlation with one another in our result. For example, *Prevotellaceae* with *RF 16*, and *Bacillaceae* with *Staphylococcaceae*, are highly correlated along with shared evolutionary lineage. Hence, these results demonstrate that there are differences in microbial interactions between CD patients and HCS. Similar differences in bacterial relationship were reflected in other sample groups.

### Detection of associations between bacterial taxa and microbial pathway

For all the bacteria with strong associations in the previous results, we identified their highly correlated KEGG orthologues (Tables 2 and 3).

**Table 2:** Identifying associations between bacterial families and their microbial pathways with KO density in Crohn’s Disease Stool

	KEGG ortholog (KO)	Module	Density
*f_Bacteroidaceae*	K02117,K02118,K02119, K02120,K02121,K02123, K02124	M00159	0.89
*f_Lachnospiraceae*	K02117,K02118,K02120, K02121 K02123,K02124	M00159	0.67

**Table 3:** Identifying associations between bacterial families and their microbial pathways with KO density in Crohn’s Disease Tissue

	KEGG ortholog (KO)	Module	Density
*f_Pseudomonadaceaef_Moraxellaceae*	K00404,K00405, K00406,K00407	M00156	0.80
*f_Pseudomonadaceae*	K01856,K03464, K01055,K03381	M00568	0.80
*f_Moraxellaceae*	K01856,K03464, K01055	M00568	0.60
*f_Pseudomonadaceaef_Moraxellaceae*	K00457,K00451, K01800,K01555	M00044	0.67
*f_Pseudomonadaceaef_Moraxellaceae*	K00166,K00167, K09699,K00253, K00249,K01968, K01969,K1376	M00036	0.62
*f_Pseudomonadaceaef_Moraxellaceae*	K02274,K02275, K02276	M00155	0.60

Several KEGG orthologues related to V/A-type H+/Na+ transporting ATPase subunit A (K02117), B (K02118), C(K02119), D(K02120), E(K02121), I(K02123), and K(K02124) showed positive correlation (Spearman’s correlation >0.6, FDR <0.05) with *Bacteroidaceae* and *Lachnospiraceae* in CDS (Table 2). These strong correlations between the abundances of bacteria taxon and gene abundances (KO) highlight genes relevant to disease phenotype in the bacterial species. A V-type ATPase in prokaryotes (M00159) KEGG module was highly associated (KO density >0.6) with above mentioned KOs in the stool samples from Crohn’s disease patients. In the CDT, Table 3 shows *Pseudomonadaceae* and *Moraxellaceae* were found to be positively correlated with several genes (KO). For those significant associations between the taxonomic clades and metagenomic gene familes, 5 strongly associated KEGG modules, viz. Cytochrome c oxidase, cbb3-type (M00156), Catechol ortho-cleavage, catechol ⇒ 3-oxoadipate (M00568), Tyrosine degradation, tyrosine ⇒ homogentisate (M00044), Leucine degradation, leucine ⇒ acetoacetate + acetyl-CoA (M00036) and Cytochrome c oxidase, prokaryotes (M00155), were identified. Additional file 2 shows the associations of all correlated bacterial genera with their highly correlated KEGG orthologues in CDT. Those three bacteria revealed strong associations with four KEGG modules, viz. Polyamine biosynthesis (M00134), Nucleotide sugar biosynthesis (M00554), PRPP biosynthesis (M00005), and Trans-cinnamate degradation (M00545).

### Split graph analysis

The resulting split graph consists of two disjoint sets of nodes, where one set corresponds to correlated microbial communities, and the other set corresponds to their microbial metabolic pathways. We automatically extracted various important components (subgraphs) from the split graphs that model the integrated network in both samples (CDS and CDT). Again, the automatic extraction of such components is implemented by finding high-weighted maximal cliques in the split graph. Due to the independence of the nodes representing the pathways, each clique in the graph contained one node representing a pathway. A high-weighted clique is the graph which will contain a group of bacteria that are highly correlated and a pathway that is highly associated or impacted by such group.

**Fig. 4:**
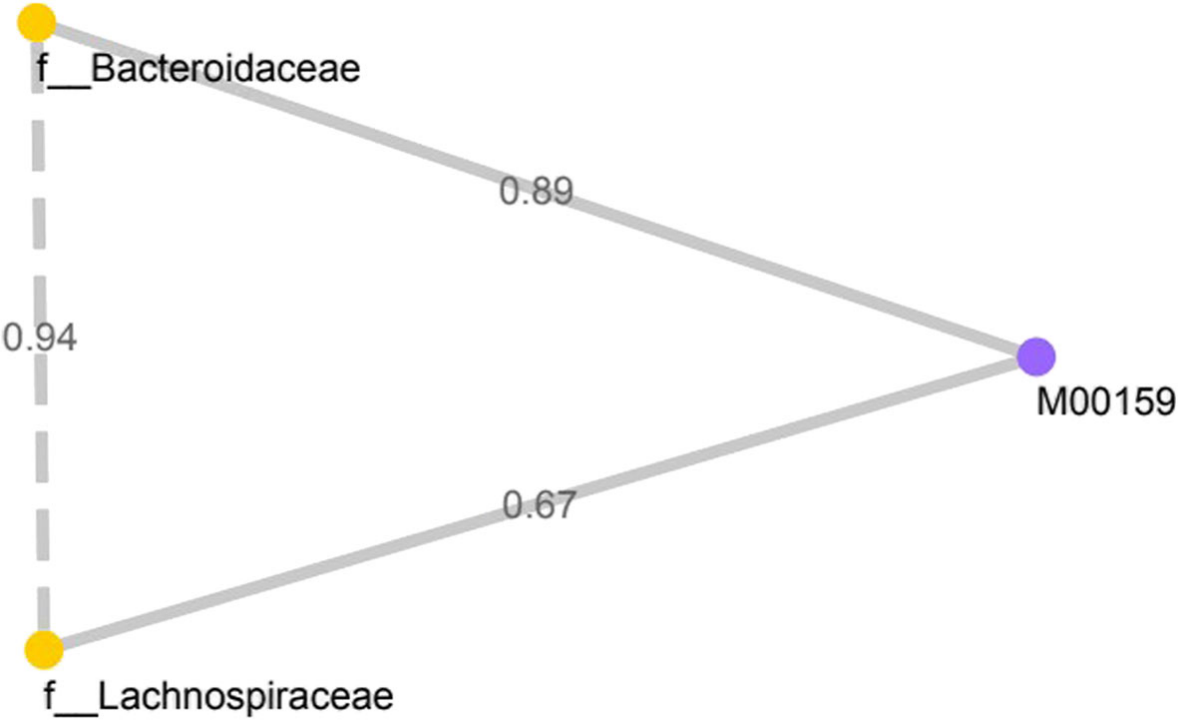
Split graph in Crohn’s disease stool samples at the family taxonomic level

**Fig. 5:**
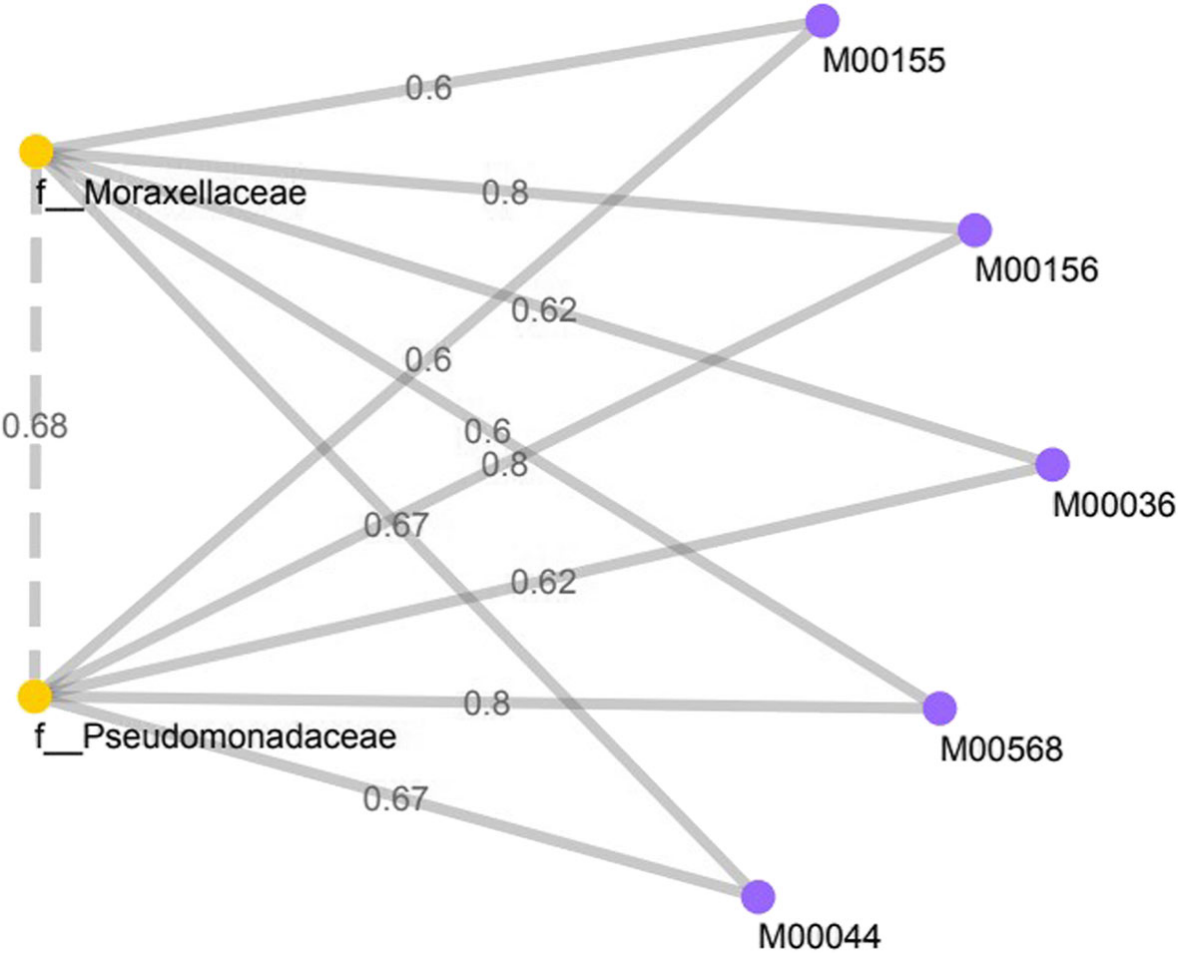
Split graph in Crohn’s disease tissue samples at the family taxonomic level

**Fig. 6:**
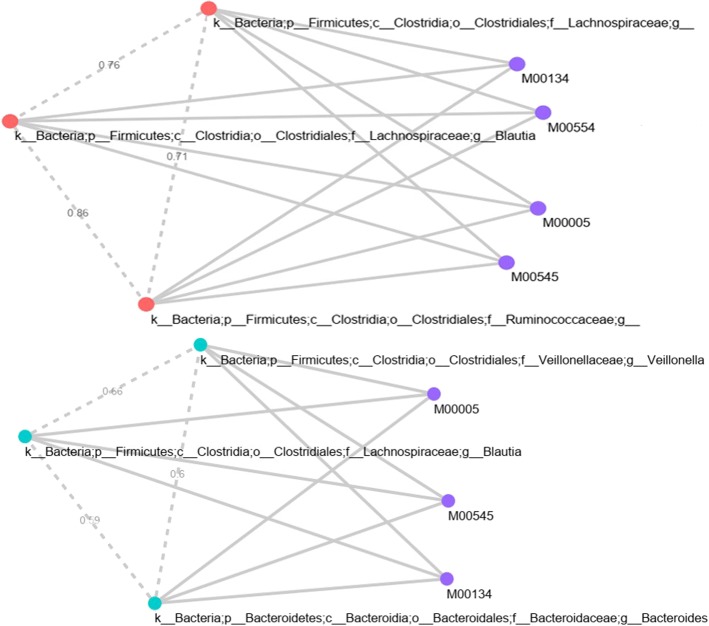
Top two split graphs in Crohn’s disease tissue samples at the genus taxonomic level

In the CDS split graph, two bacteria at the family level, *Bacteroidaceae* and *Lachnospiraceae*, are highly correlated with each other (Spearman’s correlations 0.94, FDR <0.05). This clique is associated with V-type ATPase, prokaryotes KEGG module (M00159) (Fig. 4). The quantified values for this association were weighted based upon calculating the proportions of KEGG orthologus (KO) for each correlated bacteria. Similarly, in the CDT split graph, a maximal clique of size two was identified with high correlation between *Pseudomonadaceae* and *Moraxellaceae* (Spearman’s correlations 0.68, FDR <0.05) (Fig. 5) and multiple KEGG modules were connected to this clique of bacteria (KO density >0.6). These KEGG modules in CDT are mainly involved in ATP synthesis and amino acid metabolism. Yet, the extent to which bacteria in the clique correspond to distinct functional modules in the split graph has remained largely unclear. We also extracted the split graph where the microbial components were considered at the genus level. At the genus level, we obtained multiple split graphs with different sizes of clique where each pair of bacteria are highly correlated (Spearman’s correlations >0.6, FDR <0.05) in CDT (See Additional file 3). Figure 6 represent two complete split graphs containing multiple high-weighted maximal cliques. For instance, three bacterial components correlated with PRPP biosynthesis microbial metabolic pathway constitute one of the high-weighted maximal cliques.

**Fig. 7:**
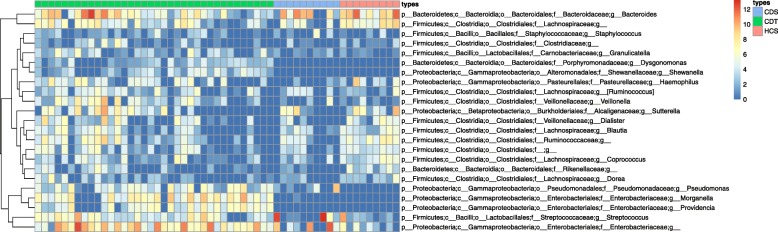
Heatmap of relative abundance of 23 bacterial genera in Crohn’s disease Stool (CDS), Crohn’s disease tissue (CDT) and control samples (HCS)

We also visualized a heatmap of OTU abundances at the genus level to assess the abundance of bacteria in the samples (Fig. 7). The abundance of *Blautia* and unknown genus from *Lachnospiraceae* family and that of unknown genus from *Ruminococcaceae* family were observed to be high in CDT samples. Likewise, the bacterial genera, *Veillonella* and *Bacteroides*, were also highly abundant in CDT group. In addition, there were no high-weighted maximal cliques obtained in split graph from CDS samples as none of the KEGG modules were significantly correlated to any of the highly correlated bacteria in CDS (Spearman’s correlation >0.6, FDR <0.05).

### Comparison of Two Population Proportions Analysis

In the split graph, the extracted high-weighted maximal cliques of microbial communities with their metabolic pathways were mostly observed in the microbiome profiles obtained mucosal tissue samples of CD patients.

Among all correlated edges in CDT and CDS networks, proportion of the correlated edges with a common family is different between CDT and CDS networks (Table 4). In other words, the proportion of correlated edges in CDT with common family is significantly different to the proportion of correlated edges in CDS with common family. Similarly, the proportion of correlated edges with common class (and phylum) is also significantly different between CDT and CDS networks.

**Table 4:** *p-value* from proportion test at different level of taxonomy

	CDS vs CDT
Family	9.467441e-10***
Order	0.3
Class	0.03*
Phylum	0.03*

Previous studies on microbiomes in Crohn’s disease revealed that fecal bacterial ecosystems differ from those in the intestinal mucosal tissue [22, 23]. Studies of microbiome in fecal samples have more challenges in identifying their community associated with respect to disease initiation and progression due to the nature of the environment. Based on these observations, we can infer that microbial dysbiosis is less tended to be shifted toward lumens in a given disease state. In order to gain a better understanding of possible microbial mechanisms, the need to examine tissue biopsies along with stool samples are highlighted.

## Discussion and conclusion

In the last couple of decades, advances in data generation and algorithmic development have highlighted the vital importance of microbiomes and the crucial role they play in impacting human health. Microbiomes are involved in many human biological processes such as modulation of the immune system, regulation of metabolic functions, and epithelial development. Given the complex and dynamic microbial communities associated with health and dysbiosis, it is imperative for understanding microbiome interactions and their relations to the host. The systems biology approach encompassing graph-theory can foster microbiome analysis and intensify our understanding of complexity in structure and functional microbial ecosystem [24]. Despite the progress in such approaches, advanced robust modeling and analytical tools are still needed to leverage the correlation between microbial and host phenotypical characteristics in order to understand significant associations between microbial community and its functional relevance.

In this work, we proposed a new approach to model such a complex set of relationships using an interesting class of graphs, called ‘Split Graphs’. Our proposed model takes advantage of its properties to capture the structure of complex microbial inter-relationships and how they contribute to the host environments. We illustrate this novel approach by examining high weighted maximal clique, containing each KEGG module and the bacterial clique, in the context of Crohn’s disease patients and healthy individuals. While deploying the model on data obtained from CD patients and healthy individuals, we were able to extract useful associations. In the CDS split graph, two highly correlated bacterial families, *Lachnospiraceae* and *Bacteroidaceae*, have been reported in previous studies to be decreased in Crohn’s disease samples and to increase the risk of disease related to intestinal inflammation [25–27]. These correlated bacterial families are associated with V-type ATPase in prokaryotes (M00159) KEGG module (Fig. 4). The disassembled form of V-ATPase can mediate vesicle leakage leading to prolonged exposure of non-sequestered monamines to monamine oxidases and then to toxic aldehydes, causing cell damage and inflammation [28, 29]. Unlike in the CDS split graphs, multiple functional KEGG modules were detected in the split graph that represents data obtained from mucosal tissue samples in CD patients. In one of the examples of the CDT split graphs, a bacterial families clique represents high correlation between *Pseudomonadaceae* and *Moraxellaceae*. In addition, multiple functional KEGG modules were found to be associated with these bacterial families. Several studies found that these two members of *Proteobacteria* were more abundant in CD sample sets [26]. It can therefore be suggested that these two taxa may be involved in a mutual relationship, with the potential role in CD pathogenesis. Furthermore, *Pseudomonadaceae* and *Moraxellaceae* were closely associated with microaerobic energy metabolism, amino acid degradation, and energy deficiency characterized by low ATP levels. These metabolic mechanisms lead to chronic inflammation that characterizes the Crohn’s disease [30, 31]. In another instance of the CDT split graphs, bacterial clique elucidates the highly correlated bacterial genera belonging to *Lachnospiraceae* and *Ruminococcaceae* families. The decreased abundance of these bacterial families have been previously known as one of the signatures of the microbial imbalances in CD patient [32, 33]. The *Marchesi et al.* study indicated the depletion of these bacteria families can be described as the disturbance of metabolic function with the observation of a lower capacity of butyrate producing of IBD microbiota [34]. Another study demonstrated that these butyrate-producing bacterial families have the capacity to improve epithelial barrier integrity as well as their butyrate production [32]. Similarly, the bacterial genera, *Veillonella* and *Bacteroides*, identified in the CDT split graph has also been reported for IBD patients. Among the commensal intestinal microbes, *Bacteroides* are found to be involved in the development of inflammation in several studies [35, 36]. Bacterial cliques identified in the CDT split graphs were highly associated with multiple metabolic functions (KEGG module). Polyamine biosynthesis, one of the modules which represents both CDT split graphs (Fig. 6), is considered to be essential for proliferation and differentiation of the renewing intestinal mucosa [37]. In several studies, the authors suggested that polyamine deficiency can be the cause of inflammation [38, 39].

The results from the LEfSe pipeline have also been identified previously as biomarkers in other studies. Some genera such as *Bacteroidetes*, *Ruminococcus*, *Pseudomonas* have been reported more frequently in CD patients [40, 41]. Among the functional biomarkers, including metabolism of glutathione biosynthesis (M00118), nitrogen metabolism (M00175), and sulfur-containing amino acid cysteine (M00338), have been reported in previous studies to be associated with Crohn’s disease [30, 42, 43].

In summary, the split graph model allowed incorporation of associations between microbial and their metabolic pathways to demonstrate significant associations while observing high correlations among the inter-bacterial relationships. Our results are consistent with existing literature. For example, we show that several microbial components and KEGG modules identified in the split graph model were also reported to be associated with Crohn’s disease patients in previous studies. In this study, we could gain valuable insights into the importance of microbial communities and their inter-relationships and also into mechanisms of how these microbial structures are correlated with different diseases such as CD. In addition, the proposed model has the ability to overlay multiple of relationships obtained from different data sources. Exploiting this particular feature of the model would be a natural next step in this line of research.

## Supplementary information

**Additional file 1** This file contains Inter-bacteria correlations in all sample groups at genus level.

**Additional file 2** This file contains associations between bacterial genera and their microbial pathways with KO density in Crohn’s disease tissue.

**Additional file 3** This file contains list of split graphs for Crohn’s disease patients from tissue group.

## Data Availability

16S rRNA datasets from NCBI SRA database with project accession number SRP039586.

## Abbreviations

CDS: Stool samples from Crohn’s disease patients
CDT: Mucosal tissue samples from Crohn’s disease patients
FDR: False discovery rate
HCS: Stool samples from healthy individuals
HUMAnN2: The HMP unified metabolic analysis network 2
IBD: Inflammatory bowel disease
KEGG: Kyoto encyclopedia of genes and genomes
KO: KEGG orthologues
KW test: Kruskal-Wallis test
LDA: Linear discriminant analysis
LEfSe: Linear discriminant analysis effect size
NCBI: National center for biotechnology information
OTU: Operational taxonomic unit (OTU)
PICRUSt: Phylogenetic investigation of communities by reconstruction of unobserved states
PRPP: Phosphoribosyl diphosphate
QIIME: Quantitative insights into microbial ecology
SparCC: Sparse correlations for compositional data
SpiecEasi: SParse InversE covariance estimation for ecological ASsociation inference
SRA: Sequence read archive
UMP: Uridine monophosphate
V-ATPase: Vacuolar ATPase
